# Rolle des Transplantationsbeauftragten

**DOI:** 10.1007/s00101-021-01023-5

**Published:** 2021-09-03

**Authors:** Barbara Sinner, Stephan Schweiger

**Affiliations:** 1 Klinik für Anästhesiologie, Universitätsklinikum Regensburg, Franz-Josef-Strauss-Allee 11, 93053 Regensburg, Deutschland; 2 Klinik und Poliklinik für Chirurgie, Universitätsklinikum Regensburg, Franz-Josef-Strauss-Allee 11, 93053 Regensburg, Deutschland

**Keywords:** Irreversibler Hirnfunktionsausfall, Organspende, Angehörigenbetreuung, Qualitätssicherung in der Gesundheitsversorgung, Rechtliche Aspekte, Irreversible loss of brain function, Organ donation, Family support, Health care quality assurance, Legal aspects

## Abstract

Alle Krankenhäuser, die nach dem Sozialgesetzbuch V als Entnahmekrankenhäuser definiert sind, sind verpflichtet, einen Transplantationsbeauftragten (TxB) zu stellen. Das Aufgabenfeld des TxB beinhaltet nicht nur die Spendererkennung, die Diagnose eines irreversiblen Hirnfunktionsausfalls sowie die Spenderevaluation und Organprotektion, sondern er begleitet den gesamten Organspendeprozess. Er verantwortet die Festlegung von innerklinischen Standards sowie die Organisation von Fort- und Weiterbildungen und ist Ansprechpartner rund um das Thema Organspende. Darüber hinaus fungiert er als Bindeglied zwischen der Koordinierungs- (Deutsche Stiftung Organtransplantation) und der Vermittlungsstelle (Eurotransplant). Seine Tätigkeit unterliegt dem Transplantationsgesetz und dessen Ausführungsgesetzen; er benötigt aber auch entsprechende Kenntnisse der verschiedenen Richtlinien zu Organspende bzw. -transplantation. Letztendlich ist der TxB auch für die Qualitätssicherung des Organspendeprozesses verantwortlich.

## Abstract (en)

All hospitals that are defined as organ donation hospitals according to the Social Act V (SGB V), are legally bound to employ a transplant coordinator (TxB). The field of activities of the TxB includes not only the identification of donors, the diagnosis of irreversible loss of brain function, donor evaluation and organ protection but also the support of the complete organ donation process. The TxB is responsible for the establishment of in-house hospital standards as well as the organization of advanced and continuing education and is the contact person for all aspects of organ donation. Furthermore, the TxB acts as a link between the coordination center (German Organ Procurement Organization) and the allocation organisation (Eurotransplant). The activities are subject to the Transplantation Act and its implementation statutes; however, the TxB also needs corresponding knowledge of the various guidelines on organ donation and transplantation. Finally, the TxB is also responsible for the quality assurance of the organ donation process.

## Einführung

Im europäischen Vergleich ist in Deutschland die Zahl der realisierten Organspenden vergleichsweise niedrig. So wurden vor Beginn der COVID-19-Pandemie im Jahr 2019 in Spanien beispielsweise 48,9 Organspenden pro 1 Mio. Einwohner durchgeführt, wohingegen in Deutschland im selben Jahr lediglich 11,2 Spenden realisiert werden konnten [1]. Verschiedene Anstrengungen werden und wurden unternommen, um die Zahl der Organspenden zu erhöhen, so u. a. im Januar 2020 der Versuch der gesetzlichen Einführung der Widerspruchslösung, die letztlich gegenüber der sog. Entscheidungslösung – eine im Wesentlichen erweiterte Zustimmungslösung – gescheitert ist. In zahlreichen Ländern konnte die Organspendebereitschaft durch die Einführung eines „Inhouse“-Koordinators oder Transplantationsbeauftragten (TxB) deutlich gesteigert werden [2]. Dabei reicht das Aufgabenfeld des TxB von der Spendererkennung, der Betreuung potenzieller Organspender, einschließlich der Feststellung des irreversiblen Hirnfunktionsausfalls (IHA), und der Organisation der erforderlichen Diagnostik bis zur Planung der Organentnahme, zusammen mit der Koordinierungsstelle Deutsche Stiftung Organtransplantation (DSO) und der Vermittlungsorganisation Eurotransplant (ET). Nicht zuletzt begleitet der TxB die Angehörigengespräche und kümmert sich im Anschluss an die Organspende um die Angehörigenbetreuung. Er unterstützt bzw. entlastet damit die behandelnden Ärzte und verbessert die Kommunikation zwischen den involvierten Einrichtungen. Er evaluiert und optimiert alle Prozesse mit Bezug auf die Organspende, für die das Entnahmekrankenhaus Verantwortung trägt. Darüber hinaus trägt er durch Information sowie regelmäßige Fort- und Weiterbildung zur höheren Akzeptanz der Organspende und damit zur Förderung derselben bei [3].

## Rechtliche Grundlagen

### Gesetzliche Grundlagen

Die Transplantationsmedizin in Deutschland wird durch verschiedene Gesetzte, Richtlinien, Verordnungen und Vorgaben geregelt. Hierzu zählen:

- Gesetz über die Spende, Entnahme und Übertragung von Organen und Geweben (Transplantationsgesetz, TPG; [4]),
- Fünftes Buch Sozialgesetzbuch (SGB V; [5]),
- Landesrecht: Gesetze der Bundesländer zur Ausführung des TPG (AGTPG), regelt u. a. Qualifikation, organisationsrechtliche Stellung etc.,
- Richtlinien (RL) der Bundesärztekammer, z. B. RL BÄK Spendererkennung [6],
- Vorgaben von Gemeinsamem Bundesausschuss (G-BA), DSO, ET etc.

Die Aufgaben des TxB sind insbesondere im § 9b des TPG geregelt [4]. Dieser sieht vor, dass die Entnahmekrankenhäuser verpflichtet sind, einen TxB zu bestellen. Die Klinik hat organisatorisch sicherzustellen, dass der TxB seine Aufgaben ordnungsgemäß erfüllen kann, und hat ihn in der Wahrnehmung seiner Aufgaben zu unterstützen. Der TxB muss von seiner klinischen Tätigkeit freigestellt werden. Der notwendige Aufwand wird vollständig refinanziert. Die Krankenhäuser müssen die Mittelverwendung nachweisen. Durch Landesrecht können die Voraussetzungen festgelegt werden, nach denen mehrere Entnahmekrankenhäuser zur Erfüllung ihrer Verpflichtung die Bestellung eines gemeinsamen TxB schriftlich vereinbaren können. Dabei ist sicherzustellen, dass der TxB seine Aufgaben in jedem der Entnahmekrankenhäuser ordnungsgemäß wahrnehmen kann. Ausnahmen können einer Genehmigung durch die zuständige Behörde unterworfen sein.

### Rechtliche Vorgaben für den Transplantationsbeauftragten

Die Tätigkeit des TxB wird durch verschiedene Gesetze geregelt (Tab. 1).

**Table 1:** 

TPG § 1a Begriffsbestimmungen	*2.* sind vermittlungspflichtige Organe die Organe Herz, Lunge, Leber, Niere, Bauchspeicheldrüse und Darm … *5.* sind nächste Angehörige in der Rangfolge ihrer Aufzählung *a)* der Ehegatte oder der eingetragene Lebenspartner *b)* die volljährigen Kinder *c)* die Eltern oder, sofern der mögliche Organ- oder Gewebespender zur Todeszeit minderjährig war und die Sorge für seine Person zu dieser Zeit nur einem Elternteil, einem Vormund oder einem Pfleger zustand, dieser Sorgeinhaber *d)* die volljährigen Geschwister *e)* die Großeltern
TPG § 3 Entnahme mit Einwilligung des Spenders	*(1)* Die Entnahme von Organen oder Geweben ist, soweit in § 4 oder § 4a nichts Abweichendes bestimmt ist, nur zulässig, wenn *1.* der Organ- oder Gewebespender in die Entnahme eingewilligt hatte *2.* der Tod des Organ- oder Gewebespenders nach Regeln, die dem Stand der Erkenntnisse der medizinischen Wissenschaft entsprechen, festgestellt ist und *3.* der Eingriff durch einen Arzt vorgenommen wird …
	*(2)* Die Entnahme von Organen oder Geweben ist unzulässig, wenn *1.* die Person, deren Tod festgestellt ist, der Organ- oder Gewebeentnahme widersprochen hatte *2.* nicht vor der Entnahme bei dem Organ- oder Gewebespender der endgültige, nichtbehebbare Ausfall der Gesamtfunktion des Großhirns, des Kleinhirns und des Hirnstamms nach Verfahrensregeln, die dem Stand der Erkenntnisse der medizinischen Wissenschaft entsprechen, festgestellt ist
TPG § 4 Entnahme mit Zustimmung anderer Personen	*(1)* Liegt dem Arzt, der die Organ- oder Gewebeentnahme vornehmen oder unter dessen Verantwortung die Gewebeentnahme nach § 3 Abs. 1 Satz 2 vorgenommen werden soll, weder eine schriftliche Einwilligung noch ein schriftlicher Widerspruch des möglichen Organ- oder Gewebespenders vor, ist dessen nächster Angehöriger zu befragen, ob ihm von diesem eine Erklärung zur Organ- oder Gewebespende bekannt ist. Ist auch dem nächsten Angehörigen eine solche Erklärung nicht bekannt, so ist die Entnahme unter den Voraussetzungen des § 3 Abs. 1 Satz 1 Nr. 2 und 3, Satz 2 und Abs. 2 Nr. 2 nur zulässig, wenn ein Arzt den nächsten Angehörigen über eine infrage kommende Organ- oder Gewebeentnahme unterrichtet und dieser ihr zugestimmt hat
	*(2)* Der nächste Angehörige ist nur dann zu einer Entscheidung nach Abs. 1 befugt, wenn er in den letzten 2 Jahren vor dem Tod des möglichen Organ- oder Gewebespenders zu diesem persönlichen Kontakt hatte
TPG § 5 Nachweisverfahren	*(1)* Feststellungen des IHA nach § 3 Abs. 1 Satz 1 Nr. 2 und Abs. 2 Nr. 2 sind jeweils durch 2 dafür qualifizierte Ärzte zu treffen, die den Organ- oder Gewebespender unabhängig voneinander untersucht haben …
	*(2)* Die an den Untersuchungen nach Abs. 1 beteiligten Ärzte dürfen weder an der Entnahme noch an der Übertragung der Organe oder Gewebe des Spenders beteiligt sein. Sie dürfen auch nicht Weisungen eines Arztes unterstehen, der an diesen Maßnahmen beteiligt ist. Die Feststellung der Untersuchungsergebnisse und ihr Zeitpunkt sind von den Ärzten unter Angabe der zugrunde liegenden Untersuchungsbefunde unverzüglich jeweils in einer Niederschrift aufzuzeichnen und zu unterschreiben …
TPG § 7 Datenverarbeitung, Auskunftspflicht	*(2)* Zur unverzüglichen Auskunft über die nach Abs. 1 erforderlichen Daten sind verpflichtet: [...]
	*6.* der Transplantationsbeauftragte des Entnahmekrankenhauses
TPG § 9a Entnahmekrankenhäuser	*1)* Entnahmekrankenhäuser sind die nach § 108 des Fünften Buches Sozialgesetzbuch oder nach anderen gesetzlichen Bestimmungen zugelassenen Krankenhäuser, die nach ihrer räumlichen und personellen Ausstattung in der Lage sind, Organentnahmen von möglichen Spendern nach § 3 oder § 4 nach Maßgabe des § 11 Abs. 4 Satz 5 zu ermöglichen …
	*2)* Die Entnahmekrankenhäuser sind verpflichtet *1.* den endgültigen, nichtbehebbaren Ausfall der Gesamtfunktion des Großhirns, des Kleinhirns und des Hirnstamms von Patienten, die nach ärztlicher Beurteilung als Organspender in Betracht kommen, nach § 5 festzustellen und der Koordinierungsstelle nach § 11 unverzüglich mitzuteilen; kommen diese Patienten zugleich als Gewebespender in Betracht, ist dies gleichzeitig mitzuteilen *2.* sicherzustellen, dass die Zuständigkeiten und Handlungsabläufe zur Erfüllung ihrer Verpflichtungen aus diesem Gesetz in einer Verfahrensanweisung festgelegt und eingehalten werden … *4.* sicherzustellen, dass das von ihnen eingesetzte medizinische Personal für seine Aufgaben qualifiziert ist … *6.* sicherzustellen, dass alle Todesfälle mit primärer oder sekundärer Hirnschädigung sowie die Gründe für eine nichterfolgte Feststellung oder für eine nichterfolgte Meldung nach Nr. 1 oder andere der Organentnahme entgegenstehende Gründe erfasst und die Daten der Koordinierungsstelle nach § 11 mindestens einmal jährlich anonymisiert übermittelt werden
TPG § 9b Transplantationsbeauftragte	*(1)* Die Entnahmekrankenhäuser bestellen mindestens einen ärztlichen Transplantationsbeauftragten, der für die Erfüllung seiner Aufgaben fachlich qualifiziert ist. Hat ein Entnahmekrankenhaus mehr als eine Intensivstation, soll für jede dieser Stationen mindestens ein Transplantationsbeauftragter bestellt werden. Der Transplantationsbeauftragte ist in Erfüllung seiner Aufgaben unmittelbar der ärztlichen Leitung des Entnahmekrankenhauses unterstellt. Er ist bei der Wahrnehmung seiner Aufgaben unabhängig und unterliegt keinen Weisungen. Die Entnahmekrankenhäuser stellen sicher, dass der Transplantationsbeauftragte seine Aufgaben ordnungsgemäß wahrnehmen kann, und unterstützen ihn dabei. Die Entnahmekrankenhäuser stellen insbesondere sicher, dass *1.* der Transplantationsbeauftragte hinzugezogen wird, wenn Patienten nach ärztlicher Beurteilung als Organspender in Betracht kommen *2.* der Transplantationsbeauftragte zur Wahrnehmung seiner Aufgaben ein Zugangsrecht zu den Intensivstationen des Entnahmekrankenhauses erhält *3.* dem Transplantationsbeauftragten zur Erfüllung seiner Verpflichtung nach Abs. 2 Nr. 5 alle erforderlichen Informationen zur Verfügung gestellt werden und *4.* durch Vertretungsregelungen die Verfügbarkeit eines Transplantationsbeauftragten gewährleistet ist Die Kosten für fachspezifische Fort- und Weiterbildungen der Transplantationsbeauftragten sind von den Entnahmekrankenhäusern zu tragen
	*(2)* Transplantationsbeauftragte sind insbesondere dafür verantwortlich *1.* dass die Entnahmekrankenhäuser ihrer Verpflichtung nach § 9a Ab. 2 Nr. 1 nachkommen *2.* dass die Angehörigen von Spendern nach § 3 oder § 4 in angemessener Weise begleitet werden *3.* die Verfahrensanweisungen nach § 9a Ab. 2 Nr. 2 zu erstellen *4.* dass das ärztliche und pflegerische Personal im Entnahmekrankenhaus über die Bedeutung und den Prozess der Organspende regelmäßig informiert wird *5.* alle Todesfälle mit primärer oder sekundärer Hirnschädigung in jedem Einzelfall, insbesondere die Gründe für eine nichterfolgte Feststellung oder eine nichterfolgte Meldung nach § 9a Abs. 2 Nr. 1 oder andere der Organentnahme entgegenstehende Gründe, auszuwerten und *6.* dass der Leitung des Entnahmekrankenhauses mindestens einmal jährlich über die Ergebnisse der Auswertung nach Nr. 5 über ihre Tätigkeit und über den Stand der Organspende im Entnahmekrankenhaus berichtet wird
	*(3)* Transplantationsbeauftragte sind so weit freizustellen, wie es zur ordnungsgemäßen Durchführung ihrer Aufgaben und zu ihrer Teilnahme an fachspezifischer Fort- und Weiterbildung erforderlich ist. Die Freistellung erfolgt mit einem Anteil von mindestens 0,1 Stellen bei bis zu je 10 Intensivbehandlungsbetten. In Entnahmekrankenhäusern, die Transplantationszentren nach § 10 Abs. 1 sind, muss die Freistellung insgesamt eine ganze Stelle betragen. Die Entnahmekrankenhäuser erhalten Ersatz für die Aufwendungen für die Freistellung der Transplantationsbeauftragten. Die zweckentsprechende Mittelverwendung ist gegenüber der Koordinierungsstelle nachzuweisen
	*(4)* Das Nähere, insbesondere zu der erforderlichen Qualifikation und organisationsrechtlichen Stellung der Transplantationsbeauftragten, wird durch Landesrecht bestimmt
TPG § 14 Datenschutz	… Anwendung der Vorschriften über den Datenschutz gemäß § 40 Abs. 1 des Bundesdatenschutzgesetzes … Die … an der Organ- oder Gewebeentnahme, der Organvermittlung oder -übertragung … beteiligten Personen … dürfen personenbezogene Daten der Spender und der Empfänger nicht offenbaren
Richtlinie gemäß § 16 Abs. 1 S. 1 Nr. 3 TPG zur ärztlichen Beurteilung nach § 9a Abs. 2 Nr. 1 TPG (RL BÄK Spendererkennung)	*I.1 Auftrag* Gegenstand dieser Richtlinie … ist die Erkennung von Patienten, die nach ärztlicher Beurteilung als Organspender in Betracht kommen (potenzielle Organspender)
	*II.1 Voraussetzung des Erkennens eines potenziellen Spenders* … Ob und welche Organe eines potenziellen Spenders sich tatsächlich zur Transplantation eignen, ist im Zweifelsfall durch das Entnahmekrankenhaus unter Hinzuziehung des Transplantationsbeauftragten und gemeinsam mit der Koordinierungsstelle zu klären
	*II.2 Verlaufsbeobachtung – klinische Symptome* Ärztliches und pflegerisches Personal sowie Transplantationsbeauftragte sollen bei beatmeten Patienten mit akuter primärer oder sekundärer Hirnschädigung auf klinische Zeichen achten, die auf einen irreversiblen Hirnfunktionsausfall hindeuten können. … Zur ärztlichen Beurteilung potenzieller Organspender ist der Transplantationsbeauftragte hinzuzuziehen (nach § 9b Abs. 1 S. 6 Nr. 1 TPG)
	*III Therapiezielfindung bei potenziellen Organspendern* … Ärztliches Personal in der Intensivmedizin sowie Transplantationsbeauftragte sollten spätestens bei unmittelbar bevorstehendem oder vermutetem irreversiblen Hirnfunktionsausfall bereits erste orientierende Gespräche („Therapie‑, Therapieziel- und Prognosegespräche“) mit den Patientenvertretern hinsichtlich einer Therapiezielfindung suchen
	*III.1 Therapie‑, Therapieziel- und Prognosegespräche* … Förderlich für das Vertrauensverhältnis ist es, wenn diese Gespräche möglichst durch einen der behandelnden Ärzte oder den Transplantationsbeauftragten geführt werden. Die Koordinierungsstelle kann unterstützend hinzugezogen werden
AGTPG	Landesrecht regelt die
	– Bestellung eines TxB durch die Krankenhäuser
	– Qualifikation des TxB
	– rechtliche Stellung des TxB innerhalb der Entnahmekrankenhäuser
	– schriftliche Vorgaben für alle in den Abauf der Spendererkennung und Organspende involvierten Personen
	– Begleitung von Angehörigen
	– Übermittlung und Qualitätssicherung aller Todesfälle durch primäre oder sekundäre Hirnschädigung an die DSO durch den TxB
	– Sicherstellung von Fortbildungen aller in den Prozess involvierten Mitarbeitenden der Entnahmekrankenhäuser

Abkürzungen: s. Abkürzungsverzeichnis

#### Stellung, Rechte und Pflichten

Der TxB ist unmittelbar an die ärztliche Leitung des Entnahmekrankenhauses angebunden. Es gilt eine Freistellungsregelung für TxB nach Maßgabe der Zahl der Intensivbetten: mindestens 0,1 Stellenanteil je 10 zu betreuende Intensivbetten, jeweils anteilig bei mehreren TxB; bei ≤ 10 Intensivbetten Wahlmöglichkeit zwischen Vergütung und Freistellung [4]. Für die Freistellung gibt es eine vollständige Vergütung der Aufwendungen der Entnahmekrankenhäuser [4]. Bei der Wahrnehmung seiner Aufgaben ist er unabhängig und unterliegt auch keinerlei Weisungen. Er besitzt ein uneingeschränktes Zugangsrecht zu allen für die Organspende relevanten Bereichen des Entnahmekrankenhauses. Auch müssen ihm alle erforderlichen Informationen durch die Krankenhausleitung zur Verfügung gestellt werden. Bei der IHA-Diagnostik durch den TxB gibt es im Übrigen weder in der RL zur Feststellung des IHA der BÄK [7] noch im TPG [4] eine Inkompatibilitätsregelung [4, 7].

Das Landesrecht des jeweiligen Bundeslandes (AGTPG) regelt die fachspezifischen Fort- und Weiterbildungen des TxB, der hierfür freigestellt werden muss. Die anfallenden Fortbildungskosten müssen durch den Krankenhausträger übernommen werden.

Der TxB ist im Gegenzug für die Erfüllung der gesetzlichen Verpflichtungen des Entnahmekrankenhauses verantwortlich [4]. Auch ist er neben anderen zur unverzüglichen Meldung jedes „severe adverse event“ (SAE) und jeder „severe adverse reaction“ (SAR) an die Koordinierungsstelle verpflichtet. Außerdem hat er eine Pflicht zur fachspezifischen Fort- und Weiterbildung (in den AGTPG geregelt).

#### Aufgaben

Zu den zentralen Tätigkeiten des TxB zählt die Identifikation potenzieller Organspender, mit anderen Worten die Erkennung, Bewertung und Prognoseabschätzung von Patienten mit primärer und sekundärer Hirnschädigung. Dies impliziert die Verantwortung für die Erfüllung der gesetzlichen Verpflichtungen – ärztliche Beurteilung und Feststellung des IHA potenzieller Organspender sowie Mitteilung an die DSO – des Entnahmekrankenhauses. Organspender müssen erkannt werden, um den Willen des Verstorbenen bezüglich einer evtl. Organspende umzusetzen. Hierzu muss der TxB bei allen Patienten mit schwerer oder infauster primärer oder sekundärer Hirnschädigung, die nach ärztlicher Beurteilung als Organspender in Betracht kommen, hinzugezogen werden [6].

Der TxB erstellt Verfahrensanweisungen für die krankenhausinterne Festlegung der Zuständigkeiten und für die Koordinierung der Handlungs- bzw. Organisationsabläufe im Bereich Organspende und Organentnahme [4]. Diese Leitlinien und Dienstanweisungen dienen zur Absicherung der Prozessqualität der Gemeinschaftsaufgabe Organspende.

Er führt regelmäßige Analysen der Todesfälle mit primärer und sekundärer Hirnschädigung (Einzelfallanalyse) unter Angabe von Gründen für eine nichterfolgte Feststellung oder eine nichterfolgte Meldung oder anderen der Organentnahme entgegenstehenden Gründen zur Qualitätssicherung durch [4]. Dies wird durch ein Softwareprogramm (TransplantCheck der DSO) unterstützt, das Verstorbene, bei denen eine möglicherweise zum IHA führende schwere Erkrankung des Gehirns verschlüsselt wurde, aus den Patientendaten nach § 21 KHEntgG herausfiltert.

Er ist verantwortlich für die klinikinterne Dokumentation über die Inzidenz von Todesfällen nach primärer und sekundärer Hirnschädigung mit möglicher Indikation zur Organspende und berichtet dies mindestens einmal jährlich an die Krankenhausleitung.

Der TxB führt regelmäßig krankenhausinterne Informationsveranstaltungen durch und steht für Aufklärungen des ärztlichen und pflegerischen Personals über die Bedeutung und den Prozess der Organspende zur Verfügung. Er ist der primäre Ansprechpartner bei Fragen zum Themenbereich Organspende und für die DSO.

Neben diesen organisatorischen Aufgaben ist der TxB in den Spendeprozess eingebunden. Neben der angemessenen Information, Begleitung und Betreuung der Angehörigen von Organspendern hat er die Aufgabe, das intensivmedizinische Personal über den gesamten Prozess einer potenziellen Organspende fachlich zu informieren und bei der Betreuung potenzieller bzw. realisierter Organspender zu unterstützen. Zudem soll er den DSO-Koordinatoren vor Ort bei der Organisation der Organentnahme helfen. Die DSO sieht im TxB den wichtigsten Ansprechpartner für die Koordinatoren. Der TxB muss bei potenziellen Spendern frühzeitig – häufig bereits vor, spätestens jedoch und dann auch verpflichtend unverzüglich nach der Feststellung des IHA – die Koordinierungsstelle (DSO) informieren, um den optimalen Ablauf des Organspendeprozesses zu gewährleisten. Selbstverständlich auch bei allen Unklarheiten bezüglich der Spendereignung sollte jederzeit die DSO hinzugezogen werden. Zu guter Letzt soll zur Erfüllung der Aufgaben des TxB das Intensivpflegepersonal auch einbezogen werden.

## Qualifikatorische Anforderungen an den Transplantationsbeauftragten

Gesetzlich ist das jeweilige Entnahmekrankenhaus für die Sicherstellung der Qualifikation des TxB verantwortlich. Von der BÄK liegt seit 2015 ein 40-stündiges, 2‑teiliges Fortbildungscurriculum vor (Teile A und B, [8]). Darüber hinaus ist der Nachweis einer begleitenden realen/virtuellen Organentnahme zu erbringen (Teil C). Voraussetzung für die Qualifikation zum TxB ist ein Facharztstatus mit ausreichender Erfahrung in der Intensivmedizin. Jedes Krankenhaus muss mindestens einen ärztlichen TxB stellen. Weitere TxB können dann auch aus Pflegekräften rekrutiert werden (in den AGTPG geregelt).

Zur Wahrnehmung seiner Aufgaben muss der TxB allerdings zusätzlich besondere Kenntnisse und Fertigkeiten der Medizin, Administration bzw. Organisation, Qualitätssicherung und Kommunikation sowie zu assoziierten juristischen und ethischen Themenbereichen erlangen [9]. Hierzu zählen die rechtlichen Grundlagen und Rahmenbedingungen, besonders das TPG und die AGTPG, sowie Leitlinien, Richtlinien oder Empfehlungen der BÄK. Ebenso muss er mit den Ausführungsbestimmungen der Vermittlungsstelle ET, den Richtlinien für die Aufnahme in die Warteliste, den Allokationsregeln und den rechtlichen Vorgaben für die Lebendspende oder Gewebespende vertraut sein [10]. Daneben sollte er die Aufgaben der Transplantationskonferenzen sowie die Voraussetzungen zur Umsetzung der geltenden Regeln kennen. Auch ein Verständnis der Zusammenarbeit der verschiedenen Organisationen wie der Koordinierungsstelle DSO, der Vermittlungsstelle ET, der Entnahmekrankenhäuser, Transplantationszentren und der verschiedenen gesundheitspolitischen Gremien (BMG, BÄK, GKV, PKV, DKG, G-BA, DTG etc.) sollte gegeben sein. Zudem ist ein vertieftes medizinisches, fachspezifisches und fachübergreifendes Wissen wie z. B. aus der Neurologie unumgänglich. Da die Identifikation potenzieller Organspender, mit anderen Worten die Erkennung und Bewertung von Patienten mit primärer und sekundärer Hirnschädigung, sowie die Prognoseabschätzung der Hirnschädigung, einschließlich ihrer Progredienz zum IHA, zu den zentralen Tätigkeiten des TxB zählen, werden besonders hier einschlägige Kompetenzen vorausgesetzt [8]. Zudem muss der TxB umfangreiche Kenntnisse, Fähigkeiten und Fertigkeiten besitzen, um den IHA erkennen, die Indikation zur IHA-Diagnostik prüfen, die klinischen Untersuchungen durchführen und dokumentieren sowie die Ergebnisse der angewandten apparativen Zusatzdiagnostik im Kontext beurteilen zu können.

Zu seinem Tätigkeitsfeld gehört auch die Spenderevaluation [4]. Neben der Bewertung von Krankheitsbildern und Risikokonstellationen für die Indikation bzw. Kontraindikation zu einer Organspende und der organspezifischen Evaluation muss auch der Empfängerschutz sorgfältige Berücksichtigung finden. Eine ggf. notwendige Zusatzdiagnostik zur besseren Beurteilung der Organfunktion und der Transplantabilität, wie z. B. eine Herzkatheter- oder CT-Untersuchung, wird vom TxB in enger Abstimmung mit dem DSO-Koordinator veranlasst. Darüber hinaus sind Kenntnisse pathophysiologischer Veränderungen im Rahmen des IHA und deren intensivmedizinische Therapie mit dem Ziel der Aufrechterhaltung der Homöostase und der Organqualität nötig. Nicht zuletzt werden vom TxB auch Führungskompetenz und kommunikative Fähigkeiten, insbesondere in der Angehörigenbegleitung, gefordert. Diese können u. a. im Curriculum Teil B „Gesprächsführung und Angehörigengespräch“ und in sog. Seminaren zur Entscheidungsbegleitung für Angehörige (EfA) erworben werden [11].

## Angehörigenbegleitung

Eine besondere Rolle spielt die Angehörigenbetreuung von Organspendern, weshalb diesem Aspekt ein eigener Abschnitt gewidmet wird. Im Kontext des Sterbens und des Todes sind Ängste, Sorgen und Hoffnungen gleichsam omnipräsent. Die Belastungen des gesamten Personals einer Intensivstation sind daher sehr hoch, nicht zuletzt, da der Prozess einer Organspende, einschließlich der IHA-Diagnostik, keine alltägliche Routine darstellt und auch niemals darstellen wird [12, 13]. Ein organspendebezogener Kontakt mit der DSO ist indiziert, wenn bei einem Patienten mit akuter primärer oder sekundärer Hirnschädigung ein unbeeinflussbar fortschreitender Verlust der Hirnstammfunktionen beobachtet wird [4]. Im Normalfall finden die Gespräche zur IHA-Diagnostik und zur potenziellen Organspende des Verstorbenen mit dem behandelnden Arzt, ggf. unter Beteiligung des Pflegepersonals, statt. Dies fällt laut TPG somit in den Verantwortungsbereich des Entnahmekrankenhauses [4]. Die behandelnden Ärzte können sich jedoch auf Wunsch vom TxB und/oder vom DSO-Koordinator unterstützen lassen [14], insbesondere bei der Eruierung des mutmaßlichen Willens des Patienten [11].

Ein Gespräch mit den Angehörigen findet auch dann statt, wenn eine Zustimmung zur Organentnahme, z. B. in Form eines Organspendeausweis oder einer Patientenverfügung vorliegt. Hier besteht eine Pflicht zur Information, einschließlich Befragung. In etwa 9 von 10 Fällen hat der Patient mit IHA zu Lebzeiten jedoch keine Entscheidung zur Organspende getroffen [14]. In enger Kooperation mit den behandelnden Intensivärzten erfolgt daher die Identifikation eines Entscheidungsträgers unter den An- bzw. Zugehörigen. Die genaue Rangfolge der Patientenvertreter – nächste Angehörige bzw. gleichgestellte Person – ist im TPG geregelt. Der Zeitpunkt des Gesprächs zur Klärung des Patientenwillens verdient besonderes Augenmerk [6]. Immer dann, wenn der IHA unmittelbar bevorsteht oder als bereits eingetreten vermutet wird, müssen das Therapieziel und damit die Indikation zu einer intensivmedizinischen Therapie im Einklang mit dem Patientenwillen überprüft werden. Als Therapieoptionen stehen in der Situation des wahrscheinlichen Todeseintritts je nach Patientenwille alternativ entweder die Fortführung der intensivmedizinischen Therapie zur Aufrechterhaltung der Organfunktionen bis zur Feststellung des IHA bzw. nach erfolgter positiver Diagnostik bis zur Realisierung der Organspende oder die Therapiebegrenzung mit Symptomkontrolle und Sterbebegleitung im Sinne der Palliation zur Auswahl. Daher muss ein Organspendewunsch des Patienten frühzeitig erkundet werden, spätestens jedoch vor Einleitung therapiebegrenzender Maßnahmen. Solange müssen intensivmedizinische Maßnahmen zur Aufrechterhaltung der Organfunktionen erfolgen. Den geeigneten Zeitpunkt und Inhalt für diese Gespräche zu finden, erfordert ein hohes Maß an Fachkompetenz, ärztlicher Erfahrung sowie kommunikativem und empathischem Vermögen. Voraussetzungen für ein gelingendes Angehörigengespräch sind eine geschützte, vertrauensvolle Gesprächsatmosphäre und ein strukturierter Gesprächsverlauf, einschließlich sorgfältiger Dokumentation, insbesondere Ablauf, Inhalt und Ergebnis sowie beteiligte Personen, z. B. mithilfe des DSO-Dokumentationsbogens [14]. Das Gespräch sollte den zurückliegenden Behandlungsverlauf, das Ergebnis, die infauste Prognose und die Notwendigkeit des IHA bzw. – wenn bereits eingetreten – die unmissverständliche Mitteilung des IHA sowie das Anliegen der Organ- und Gewebespende, einschließlich der bis zur Realisierung nötigen, ggf. erweiterten intensivmedizinischen Maßnahmen, beinhalten. Dies impliziert Abwägungen zwischen dem Organspendewunsch und dem Willen zur Therapiebegrenzung. Im Gespräch sollten auch die erforderlichen Abläufe und die Möglichkeiten der Abschiednahme thematisiert werden. Es sollte fachkompetent, ergebnisoffen und empathisch geführt werden. Bei der Eruierung des mutmaßlichen Patientenwillens spielen Aspekte wie Entscheidungen am Lebensende mit Therapiezieländerung im Kontext von Patientenverfügung, Vorsorgevollmacht, Betreuung und Organspendeausweis eine gewichtige Rolle, die eine Unterstützung durch den TxB aufgrund seiner spezifischen Kompetenz sinnvoll erscheinen lässt [6, 11]. Die Angehörigen sollen eine Entscheidung nach dem mündlichen bzw. mutmaßlichen Willen des potenziellen Organspenders oder im Fall eines nichteruierbaren Willens nach ihren eigenen Wertvorstellungen treffen. Diese Entscheidung muss in jedem Falle respektiert und darf nicht bewertet werden. Der TxB zusammen mit dem behandelnden Arzt kann den Familienmitgliedern umfassende Informationen zum Thema Organspende und Transplantation liefern und sie bei der Entscheidungsfindung begleiten, um eine für alle tragbare und langfristig stabile Entscheidung im Sinne des Verstorbenen zu treffen und so Nachentscheidungsdissonanzen zu vermeiden.

## Umsetzung der Rechtsvorschriften am Universitätsklinikum Regensburg

### Implementierung des Transplantationsbeauftragten

Um die hausinternen Abläufe rund um die Organspende zu optimieren, wurde an am Universitätsklinikum Regensburg (UKR) eine Reihe von Maßnahmen im Rahmen der Implementierung des TxB ergriffen; diese werden im Folgenden beispielhaft vorgestellt:

- Da das UKR mehrere Intensivstationen unterhält, wurde auf den relevanten Intensivstationen je ein TxB benannt, der sich in Teilzeit (Stellenanteil 0,25) u. a. um die Spenderevaluation vor Ort kümmert. Unterstützt wird das TxB-Team (eine Oberärztin und 3 Oberärzte, in der Summe eine Vollkraftstelle) durch eine Ärztin der Stabsabteilung Qualitätsmanagement.
- Es wurde ein 24/7-Rufdienst mit Konsiliarfunktion für den TxB eingerichtet, sodass rund um die Uhr ein kompetenter Ansprechpartner in Fragen der Organspende für die Intensivstationen und die interdisziplinäre Notaufnahme zur Verfügung steht.
- Um für den Bereich Organspende und Organentnahme Schnittstellen bzw. Zuständigkeiten zu klären und Handlungsabläufe zu optimieren, wurden eine hausinterne „standard operating procedure“ (SOP) „IHA und Organspende“ sowie ein Ablaufschema „Meldung bei schwersten Schädigungen der Hirnfunktion“ zur Evaluation einer potenziellen Organspende erarbeitet und in den relevanten Arbeitsbereichen bekanntgegeben sowie im Qualitätsmanagementdokumentationssystem „roXtra“ hinterlegt.
- Es werden regelmäßige Fort- bzw. Weiterbildungen für die Mitarbeitenden (ärztliches und pflegerisches Personal) der Intensivstationen und der interdisziplinären Notaufnahme durch die TxB veranstaltet.
- Zur Qualitätssicherung der Identifikation potenzieller Organspender werden zusammen mit den relevanten Abteilungen und einer regionalen Vertreterin der DSO 4‑ bis 6‑mal/Jahr sog. „Qualitätszirkel Organspende“ abgehalten. Hierbei erfolgen retrospektive Einzelfallanalysen, die im Vorfeld durch den leitenden TxB zusammen mit der Ärztin des Qualitätsmanagements mithilfe eines Programms zur Selektion von Daten Verstorbener mit einer relevanten Hirnschädigung (DSO-TransplantCheck) vorbereitet wurden [15]. Im Nachgang erfolgt die klinikinterne Dokumentation der Inzidenz von Todesfällen nach primärer und sekundärer Hirnschädigung mit möglicher Indikation zur Organspende, ebenfalls im Programm TransplantCheck.
- Nach der statistischen Auswertung aller potenziellen und realisierten Organspenden erfolgt durch den leitenden TxB ein jährlicher Bericht an die Krankenhausleitung und an den „Lenkungsausschuss Transplantationsmedizin“.
- Beratung und Unterstützung des behandelnden Intensivteams während des gesamten Organspendeprozesses, auch über den Einsatz ggf. supportiver organprotektiver Maßnahmen zur Spenderkonditionierung.
- Kontaktpflege zur DSO im Allgemeinen und insbesondere im Vorfeld einer potenziellen Organspende sowie Unterstützung der DSO-Koordinatoren vor Ort bei der Organisation der Organentnahme durch die TxB.
- Bei Wunsch des Behandlungsteams Aufklärung, Begleitung und Betreuung der An- bzw. Zugehörigen von Organspendern durch den TxB.
- Einzelne Intensivpflegekräfte wurden nach entsprechender Fortbildung für die Aufgabenerfüllung der TxB miteingebunden.

### Komplexer Fall einer Organspendeevaluation

Um die Rolle des TxB bei der Organspenderdetektion, -evaluation und -selektion anschaulicher darzustellen, wird im Folgenden über einen realen, sehr außergewöhnlichen Fall aus dem UKR berichtet. Dieser Fall ist ausreichend komplex, um verschiedenste Aspekte im Prozess der Ermittlung potenzieller Organspender herauszuarbeiten sowie die sich daraus ergebenden Aufgaben und die nötigen Kompetenzen vonseiten des TxB aufzuzeigen.

Es handelt sich um einen 39-jährigen Patienten, der sich zur Evaluation einer Herztransplantationslistung aufgrund erneuter kardialer Dekompensation bei ischämischer Kardiomyopathie mit mehrfachen Koronarinterventionen und residueller Mitralklappeninsuffizienz nach bereits 2‑maliger interventionellen Mitralklappenreparatur (sog. MitraClip) im Hause befand. Im Anschluss an einen frustranen Versuch, einen ZVK in der rechten V. subclavia anzulegen, der aufgrund der Zustandsverschlechterung des Patienten nötig wurde, kam es zur respiratorischen Verschlechterung mit passageren Hämoptysen und peripheren Sauerstoffsättigungseinbrüchen. Bei V. a. einen rechtsseitigen Hämatopneumothorax im Thoraxröntgenbild erfolgte umgehend eine Thoraxdrainagenanlage, aus der sich Blut entleerte, sodass die Indikation zur notfallmäßigen Thorakotomie gestellt wurde. Noch vor Beginn der Operation wurde der Patient jedoch aufgrund einer elektromechanischen Entkopplung reanimationspflichtig, sodass eine venoarterielle extrakorporale Membranoxygenierung (va-ECMO) unter kardiopulmonalen Reanimationsbedingungen angelegt werden musste. Danach wurden das intrathorakale Hämatom unter Massivtransfusion ausgeräumt und der Thorax bei nichtidentifizierbarer Lokalisation der Blutungsquelle vorerst austamponiert. Auf der Intensivstation wurde wegen der zunehmend unzureichenden Oxygenierung, bestätigt durch Blutgasanalysen und zerebrales NIRS-Monitoring, auf eine vva-ECMO eskaliert. Darunter besserte sich der Gasaustausch deutlich. Der anfänglich laminare arterielle Blutfluss mit konsekutiv nötigen intermittierenden manuellen Thoraxkompressionen zur Vermeidung intrakardialer Thromben ging unter differenzierter Volumen- sowie positiv-inotroper Therapie rasch wieder in einen pulsatilen Blutfluss über. Die kardiopulmonale Situation ließ sich zunehmend stabilisieren, sodass der Patient zuletzt keiner medikamentösen hämodynamischen Unterstützung mehr bedurfte und der vva-ECMO-Support reduziert werden konnte. Unter der hypothetischen Annahme, es würde unter va-ECMO weiterhin ein kardiogener Schock bestehen, wären eine IHA-Diagnostik und insbesondere der Apnoetest kaum durchführbar gewesen, da eine der Voraussetzungen (Ausschluss eines Kreislaufschocks) für die klinische IHA-Diagnostik nicht gegeben und der Apnoetest bei vollständiger Abhängigkeit von der va-ECMO nicht durchführbar gewesen wäre. Anstelle des nichtdurchführbaren Apnoetests hätte der obligate Nachweis eines zerebralen Zirkulationsstillstandes mithilfe der Perfusionsszintigraphie erbracht werden müssen, da eine CT-Angiographie und Doppler‑/Duplexsonographie bei laminarem Blutfluss undurchführbar sind. Insgesamt gilt jedoch eine valide IHA-Diagnostik bei va-ECMO mit laminarem Flussprofil in der Fachliteratur als strittig, sodass aufgrund des momentanen Wissensstandes eher davon abgeraten wird [16]. Anders verhält es sich, wenn, wie bei dem oben beschriebenen Fall, der Patient unter va-ECMO eine kardiale Restfunktion mit pulsatilem Fluss aufweist. Hierbei kann die IHA-Diagnostik unter Berücksichtigung bestimmter Regeln und unter Beachtung eines „Harlekin-Syndroms“ mit variabler Mischzone („Wasserscheide“) und konsekutiv ungleicher CO_2_-Spannung am rechten und am linken Glomus caroticum relativ unproblematisch durchgeführt werden.

Post reanimationem stellte sich im Verlauf ein akutes Nierenversagen (AKI, KDIGO-Stadium 3) mit zunehmend steigenden Nierenretentionsparametern und abnehmender, zuletzt auch sistierender Diurese ein. Die leberspezifischen Laborparameter (Transaminasen, Bilirubin) bei chronischer Hepatitis B (HBsAg+/Anti-HBc+/HBV-DNA [PCR]+) waren leicht erhöht. Zudem stand bei im Vorfeld positivem Tuberkulose(TBC)-ELISPOT im Blut und Nachweis einer lymphogranulozytäre Entzündung im Kolon der Verdacht auf eine TBC mit Darmbeteiligung im Raum. Eine SARS-CoV‑2 Infektion konnte ausgeschlossen werden.

Unter frühzeitiger Hinzuziehung des Transplantationsbeauftragten wurde das weitere Prozedere festgelegt [4]. Bei vermuteter hypoxischer Hirnschädigung unklaren Ausmaßes nach kardiopulmonaler Reanimation erfolgte am folgenden Tag eine cCT-Kontrolle, die beginnende Zeichen eines hypoxischen Hirnschadens erkennen ließ. Die NSE-Serum-Werte waren im Verlauf ebenfalls stark erhöht (max. 262 µg/l). Weiterhin war im EEG keinerlei kortikale elektrische Aktivität, ein sog. Nulllinien-EEG, nachweisbar. Eine Aufwachreaktion trotz ausreichend langer Absetzung der Analgosedation blieb ebenfalls aus. Der Patient hatte aber weiterhin in der neurologischen Untersuchung noch einen spontanen Atemantrieb sowie einen Kornealreflex. In der Zusammenschau der Befunde war aus fachneurologischer Sicht somit eine sehr limitierte Prognose bei Z. n. zerebraler Hypoxie zu erwarten.

Aufgrund des unmittelbar bevorstehenden IHA muss der Patientenwillen (schriftlich, mündlich, mutmaßlich) eruiert werden. Es existierte weder eine Patientenvorausverfügung oder -vollmacht noch ein Organspendeausweis. Bei erwartbarer Progredienz der Hirnschädigung zum IHA wurde vom TxB erstmalig Kontakt zur DSO aufgenommen, um bereits im Vorfeld die prinzipielle Eignung als Organspender (in diesem Fall Nieren, Leber) bei marginaler Organfunktion und den oben genannten Vorerkrankungen abzuklären. Die übrigen Organe (Herz, Lungen Pankreas, Dünndarm) schieden aufgrund des oben skizzierten Krankheitsverlaufes von vornherein aus. Die Nieren hatten zu diesem Zeitpunkt noch eine ausreichende Stundendiurese bei erhöhten Nierenretentionsparametern im Sinne eines AKI-KDIGO-Stadiums 2. Nach Rücksprache mit dem zuständigen DSO-Koordinator wurde vereinbart, dass eine Organspende der Nieren bzw. der Leber trotz akut eingeschränkter Funktion zum aktuellen Zeitpunkt nicht ausgeschlossen sei. Allerdings müsste der V. a. eine Tuberkulose vonseiten der Infektiologen nach entsprechender Diagnostik mit hinreichender Sicherheit ausgeschlossen werden. Eine chronische Hepatitis B stellt durch die erweiterten Spenderkriterien der BÄK keine absolute Kontraindikation mehr für eine Transplantation dar [17].

Somit wurde mit dem behandelnden Intensivteam vereinbart, dass die intensivmedizinischen Maßnahmen bis zur endgültigen Festlegung des im Einklang mit dem Patientenwillen formulierten Therapieziels fortzuführen seien (bridge-to-decision). Insbesondere sollte das Thema Organspende bei den Angehörigen angesprochen worden sein, da ein Organspendewunsch frühzeitig, spätestens jedoch vor Einleitung therapiebegrenzender Maßnahmen, ermittelt werden muss [6]. Eine Teilnahme an dem Gespräch mit den Angehörigen zur Klärung des Patientenwillens, einschließlich des Organspendewunsches, sowie eine evtl. weitere Begleitung der Angehörigen wurde dem Intensivteam vom TxB angeboten [4]. Zur Ermittlung des Patientenwillens musste der Patientenvertreter – in diesem Fall die Ehefrau – befragt und nur bei nichtmöglicher Feststellung des Patientenwillens in Bezug auf die Organspende sollten die eigenen Wertvorstellungen des Stellvertreters herangezogen werden. Folgende Therapieziele standen zum damaligen Zeitpunkt zur Disposition:

- Weiterführung der Therapieanstrengungen bei äußerst schlechter Prognose aufgrund des Willens des Patienten,
- Zulassen des Sterbens mit Symptomlinderung („best supportive care“) und Sterbebegleitung aufgrund des Willens des Patienten nach negativer Klärung der Organspendeoption,
- Fortführung der bisherigen intensivmedizinischen Maßnahmen bis zur endgültigen Feststellung des IHA nach positiver Klärung der Organspendeoption sowie anschließende überbrückende Organprotektion bis zur Organspende [6].

Weiterhin wurde eine infektiologische Stellungnahme bezüglich der Wahrscheinlichkeit einer Tuberkuloseinfektion eingeholt. Bei aktuell aufgrund der mittlerweile vorliegenden Befunde (Darmbiopsie, Endotrachealsekrete, Aszites etc.) zwar nur sehr geringem V. a. eine TBC hätte diese laut den Infektiologen jedoch erst nach ausreichend langer (ca. 4-wöchiger) Bebrütung der endotrachealen Kulturen mit Sicherheit ausgeschlossen werden können. Dies wäre auf eine Einzelfallentscheidung mit Nutzen-Risiko-Abwägung hinausgelaufen, da es hierfür kein standardisiertes Vorgehen gibt. Die Organspende wurde allerdings bereits eindeutig nach einem ausführlichen Gespräch mit der Familie durch den betreuenden Intensivarzt, nachdem der Wille des Patienten nicht eruiert werden konnte, aufgrund der eigenen Wertvorstellung der Ehefrau abgelehnt [4]. Es wurde daraufhin nach dem mutmaßlichen Willen des Patienten eine Sterbebegleitung unter Beisein der Familie eingeleitet. Die DSO wurde abschließend vom TxB über den Ausgang informiert. Aufgrund der ungeklärten Todesursache bei schwerwiegender, den üblichen Rahmen übersteigender iatrogener Komplikation mit Todesfolge wurden die Ermittlungsbehörden (zuständige Polizeidienststelle, Staatsanwaltschaft) informiert. Bei positivem Organspendewunsch sowie in absehbarer Zeit erwartbarer Progredienz zum IHA wären mit Einwilligung des Spenders bzw. seines Stellvertreters, in diesem Fall der Ehefrau, die bisherigen intensivmedizinischen Maßnahmen bis zur endgültigen Feststellung des IHA fortzuführen gewesen. Die gerade noch akzeptable Wartezeit, in der eine Progredienz bis zum IHA erwartet werden darf, muss im Einvernehmen mit den Angehörigen/dem Patientenvertreter vereinbart werden [18]. Bei vermutetem IHA würde dann durch den TxB zusammen mit einem neurochirurgischen/neurologischen Facharzt unabhängig voneinander eine klinische IHA-Diagnostik, einschließlich Irreversibilitätsnachweis, nach den Maßgaben der BÄK durchgeführt [7]. Nach endgültiger Feststellung des Todes wäre rechtlich noch die Freigabe zur Organentnahme durch die Ermittlungsbehörden notwendig geworden. Von der Diagnostik des IHA bis zur Explantation wären organprotektive Überbrückungsmaßnahmen bridge to explant) zur optimalen Spenderkonditionierung – immer nur mit Einwilligung des Spenders (vorausverfügt) oder seines Stellvertreters – indiziert sowie neben der Anamnese und körperlichen Untersuchung die notwendigen Labor- und apparativen Untersuchungen zur Spenderdiagnostik in Absprache mit der DSO zu veranlassen gewesen [7].

## Resümee

Die dargestellten vielfältigen Funktionen des TxB erfordern entsprechende Erfahrung, Kenntnisse und Fähigkeiten, die sowohl eine Professionalisierung als auch ein großes Engagement und stetige Lernbereitschaft voraussetzen. Er kann entscheidend zur Aufklärung bei den Themenbereichen IHA und Organspende und somit zu mehr Vertrauen und Transparenz beitragen. Ist die Institution TxB als Schlüsselfigur rund um die Organspende in den Entnahmekrankenhäusern erst einmal etabliert, ist eine der organisatorischen Rahmenbedingungen geschaffen, um die Organspende in Deutschland weiter zu befördern. Die Möglichkeit einer Organspende könnte dann zukünftig als ein selbstverständlicher Bestandteil des „end of life treatment“ angesehen werden.

## Fazit für die Praxis

- Der Transplantationsbeauftragte (TxB) unterliegt gesetzlichen Vorgaben und Bestimmungen.
- Er ist vom Entnahmekrankenhaus zu stellen. Seine Qualifikation muss sichergestellt werden.
- Er koordiniert sämtliche im Organspendeprozess notwenigen Schritte von der Spendererkennung über die Diagnostik des irreversiblen Hirnfunktionsausfalls (IHA) bis zur Spenderevaluation.
- Er ist für die Fort-und Weiterbildung bezüglich der Organspende innerhalb des Krankenhauses verantwortlich.
- Er legt Verfahrensanweisungen für die Spendererkennung und den gesamten Organspendeprozess fest und ist für die Qualitätssicherung des gesamten Spendeprozesses, u.a. mittels retrospektiver Todesfallanalysen verantwortlich.
- Er ist für die Mitbetreuung der An- bzw. Zugehörigen in Zusammenarbeit mit dem Intensivteam und der Deutschen Stiftung Organtransplantation (DSO) zuständig.

## Abkürzungen

AGTPG: Gesetz zur Ausführung des Transplantationsgesetzes
AKI: Akutes Nierenversagen
BÄK: Bundesärztekammer
BMG: Bundesministerium für Gesundheit
cCT: Kraniale Computertomographie
COVID-19: „Coronavirus disease 2019“
DKG: Deutsche Krankenhausgesellschaft
DSO: Deutsche Stiftung Organtransplantation
DTG: Deutsche Transplantationsgesellschaft
EfA: Entscheidungsbegleitung für Angehörige
ELISPOT: „Enzyme linked immunospot“
ET: Eurotransplant
G‑BA: Gemeinsamer Bundessausschuss
GKV: Gesetzliche Krankenversicherungen
HB: Hepatitis B
HBV: Hepatitis-B-Virus
IHA: Irreversibler Hirnfunktionsausfall
KDIGO: Kidney Disease: Improving Global Outcomes
KHEntgG: Krankenhausentgeltgesetz
NIRS: Nah-Infrarot-Spektroskopie
NSE: Neuronenspezifische Enolase
PCR: „Polymerase chain reaction“
PKV: Private Krankenversicherungen
RL: Richtlinie
SAE: „Severe adverse event“
SAR: „Severe adverse reaction“
SARS-CoV‑2: „Severe acute respiratory syndrome coronavirus 2“
SGB: Sozialgesetzbuch
TBC: Tuberkulose
TPG: Transplantationsgesetz
TxB: Transplantationsbeauftragte/Transplantationsbeauftragter
va-ECMO: Venoarterielle extrakorporale Membranoxygenierung
ZVK: Zentraler Venenkatheter
